# Central autonomic dysfunction in multiple system atrophy: can we measure it with MRI?

**DOI:** 10.1007/s10286-020-00695-0

**Published:** 2020-05-16

**Authors:** Thilo van Eimeren

**Affiliations:** 1 Department of Neurology, University of Cologne, Cologne, Germany; 2 Department of Nuclear Medicine, University of Cologne, Cologne, Germany; 3 German Center for Neurodegenerative Diseases (DZNE), Bonn-Cologne, Germany

Multiple system atrophy (MSA) is an atypical parkinsonian disorder characterized by severe efferent baroreflex failure (i.e., cardiovascular autonomic failure) resulting in neurogenic orthostatic hypotension and supine hypertension [4–6]. Imaging studies with cardiac ^123^I-metaiodobenzylguanidine (MIBG) have shown that, in contrast to Parkinson disease, cardiac sympathetic postganglionic innervation is largely intact in patients with MSA [9]. This, in addition to additional pathological and biochemical evidence reviewed elsewhere [3], indicates that efferent baroreflex failure in MSA is a consequence of central, rather than peripheral, autonomic neurodegeneration. The central autonomic network is complex [2]. The main regulatory pathways converge in the brainstem, especially in the medulla oblongata (MO), with the vagus nuclei as the main parasympathetic outlet and the rostral ventrolateral medulla as the main area of sympathetic control. Currently, there is no cure for MSA. Novel therapies in the pipeline hold great promise to dramatically improve the prognosis of MSA if the intervention is early enough [7]. However, inclusion of MSA patients in clinical trials early in the disease course is problematic since accurate diagnosis can be difficult at this stage. Due to the timely demand for a more systematic description of utility in the field of neuroimaging biomarkers in atypical parkinsonian disorders, including MSA, a recent proposal has been made to address these pending issues [10]. In sum, neuroimaging is a powerful tool with great utility for both: the unveiling of neuropathological mechanisms of disease, and the identification of markers which increase the likelihood of a condition (i.e., diagnostic biomarkers).

In this issue of *Clinical Autonomic Research*, Suzuki and colleagues retrospectively investigated the relationship between brainstem and MO diameter as a proxy measure of cardiovascular autonomic dysfunction in patients with MSA in comparison to healthy controls [8]. They report a correlation between the midsagittal sub-pontine MO diameter and a hybrid score of parasympathetic dysfunction, while the sympathetic score was correlated with the short diameter of an ellipsoid placed in the midsagittal midbrain. The main conclusion of the authors is that “the anteroposterior diameter of the MO is a potential imaging marker of parasympathetic dysfunction in MSA”. The study has a number of strengths. Maybe the greatest is the pragmatic methodological approach of a landmark-based diameter, which can be easily and quickly assessed in clinical practice. Even the number of different and heterogeneous scanners used in the study (4 in total) could be considered a strength, since the applicability to other systems in other settings is more credible than if all participants would have been investigated with a single particular machine. Although the authors did not perform a normalization of the measures by total intracranial volume (as a proxy for brain size), which is a standard in these kind of quantitative neuroimaging assessments, this strategy arguably serves the purpose of clinical usability better than a more elaborated and complex method.

There are a number of minor technical limitations of the study; however, the main problem lies in its utility. Assuming that the MO diameter is a potential marker of parasympathetic dysfunction—would anyone use prefer to quantify parasympathetic dysfunction, instead of cheaper, non-invasive cardiovascular autonomic testing methods already available? Suzuki and colleagues did not tell us whether the MO diameter has higher accuracy or better sensitivity to detect parasympathetic dysfunction. Hence, the diagnostic utility cannot readily be inferred. But what about the ability to unveil neuropathological mechanisms of cardiovascular autonomic dysfunction? The precise delineation of the structural correlates underlying autonomic dysfunction in MSA would certainly be of great value. However, the authors limited their analysis only to particular diameters, which are not hypothesis driven, but determined by their anatomical prominence as a landmark. The authors themselves concede that they “need to consider more optimal evaluation methods”. As to which methods would be more optimal and when these methods would be implemented is left open at this point.

Despite the relatively crude methodology, the results of the study are still interesting from a pathophysiological viewpoint. First of all, the fact that sympathetic and parasympathetic scores show distinguishable structural correlates is intriguing. Moreover, a major role of midbrain atrophy in autonomic dysfunction in general and sympathetic dysfunction in particular may be considered a fair bit surprising. On the other hand, midbrain diameter may shrink in MSA as a consequence of Wallerian degeneration of hypothalamic or paraventricular neurons. If so, a pronounced correlation with sympathetic functions may point to a more rostral point of origin than the midbrain. The result that the rostral, but not the caudal MO diameter was correlated with parasympathetic function is less surprising, since the vagal nuclei are positioned more in the rostral than in the caudal MO. Conversely, the fact that rostral MO diameter did not correlate well with sympathetic functions is astonishing. After all, the pathology of the rostral MO has been shown to be associated with sympathetic dysfunction in MSA [1]. All in all, the study of Suzuki and colleagues is certainly interesting and prompts critical questions. However, one cannot help but ponder on the many insights that a “more optimal evaluation method” may have generated.
